# Supplementary material for: Discordance between gut-derived appetite hormones and energy intake in humans
Source: medRxiv. 2023 Oct 19:2023.05.10.23289718. Originally published 2023 May 11. Preprint. [Version 2] doi: 10.1101/2023.05.10.23289718 (PMC10327278; doi:10.1101/2023.05.10.23289718)
Supplement: Supplement 1 [file NIHPP2023.05.10.23289718v2-supplement-1.pdf]

medRxiv preprint doi: https://doi.org/10.1101/2023.05.10.23289718; this version posted October 19, 2023. The copyright holder for this preprint (which was not certified by peer review) is the author/funder, who has granted medRxiv a license to display the preprint in perpetuity.
This article is a US Government work. It is not subject to copyright under 17 USC 105 and is also made available for use under a CC0 license.

SUPPLEMENTAL INFORMATION

Discordance between gut-derived appetite hormones and energy intake in humans

Aaron Hengist, Christina M. Sciarrillo, Juen Guo, Mary Walter, Kevin D. Hall

This document contains 1 supplemental figure and 1 supplemental table.

medRxiv preprint doi: https://doi.org/10.1101/2023.05.10.23289718; this version posted October 19, 2023. The copyright holder for this preprint (which was not certified by peer review) is the author/funder, who has granted medRxiv a license to display the preprint in perpetuity. This article is a US Government work. It is not subject to copyright under 17 USC 105 and is also made available for use under a CC0 license.

Figure S1. Gut hormone responses sub-divided into body mass index categories. Data are mean ± SEM and individual responses. n=20. *p*-values from two-way ANOVA of diet by BMI category.
(A) Participants split by BMI category
(B) Total energy intake
(C) active glucagon-like peptide-1 (GLP-1)
(D) total glucose-dependent insulinotropic polypeptide (GIP)
(E) peptide YY (PYY)
(F) leptin
(G) total ghrelin
(H) active ghrelin

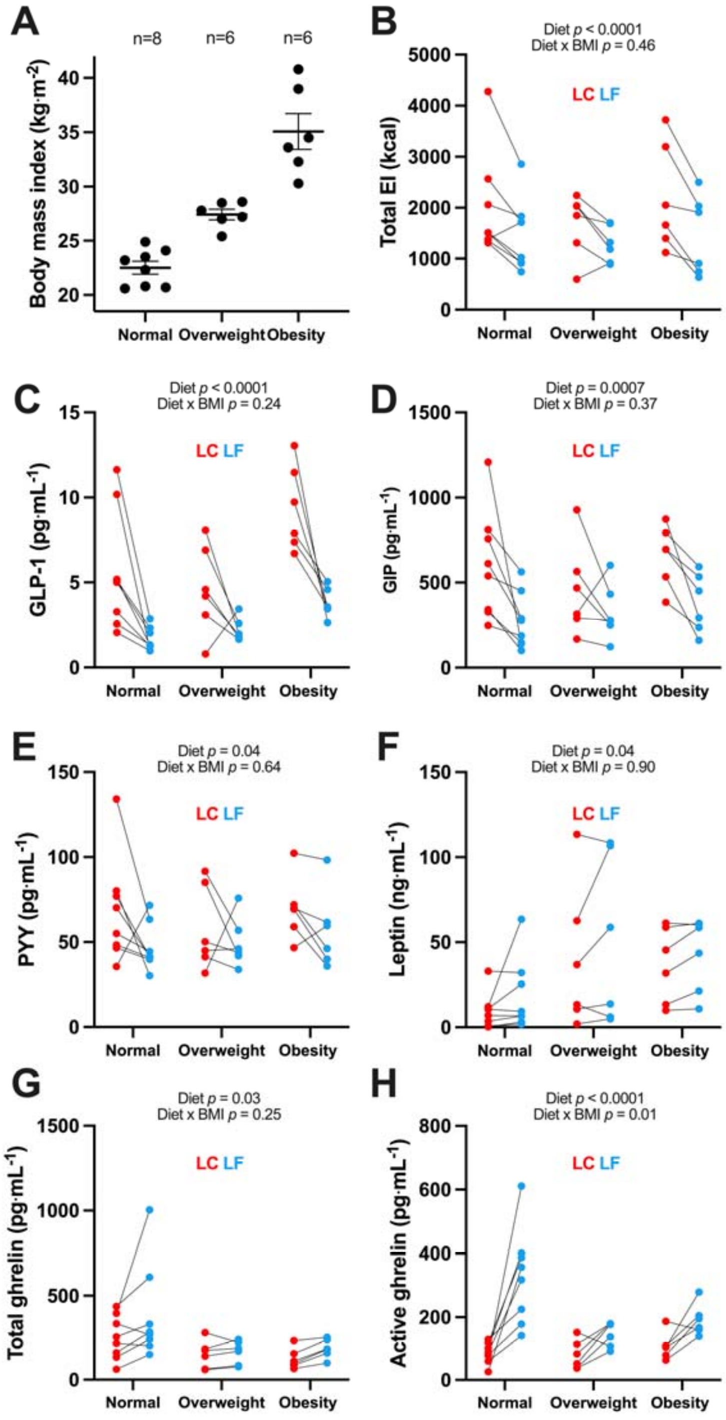

medRxiv preprint doi: https://doi.org/10.1101/2023.05.10.23289718; this version posted October 19, 2023. The copyright holder for this preprint (which was not certified by peer review) is the author/funder, who has granted medRxiv a license to display the preprint in perpetuity.
This article is a US Government work. It is not subject to copyright under 17 USC 105 and is also made available for use under a CC0 license.

Table S1. Correlations between mean postprandial responses to isocaloric low carbohydrate (LC) or low fat (LF) meals following habituation to each diet in a randomized crossover design with the subsequent mass intake in the lunch meal and total intake across the day following the test meal.

	Subsequent **lunch** mass intake during test meal day		Subsequent **total** mass intake during test meal day	
Mean postprandial concentrations	LC	LF	LC	LF
Active GLP-1	-0.11 (0.63)	-0.21 (0.37)	-0.12 (0.62)	-0.14 (0.55)
Total GIP	-0.20 (0.39)	-0.06 (0.79)	-0.15 (0.54)	0.22 (0.36)
PYY	-0.22 (0.36)	0.07 (0.76)	-0.33 (0.15)	0.10 (0.68)
Total ghrelin	-0.30 (0.20)	0.44 (0.0504)	-0.06 (0.82)	0.38 (0.10)
Active ghrelin	0.004 (0.99)	0.35 (0.13)	0.06 (0.81)	0.20 (0.39)
Leptin	-0.06 (0.81)	**-0.48 (0.03)**	-0.07 (0.76)	-0.40 (0.08)

Data are Pearson's r (p-value). n=20.
